# Evans Syndrome in the Context of Incomplete Systemic Lupus Erythematosus

**DOI:** 10.7759/cureus.25795

**Published:** 2022-06-09

**Authors:** Sarah Abu Kar, Amandeep Kaur

**Affiliations:** 1 Internal Medicine, Richmond University Medical Center, Staten Island, USA; 2 Hematology and Oncology, Richmond University Medical Center, Staten Island, USA

**Keywords:** evans syndrome, autoimmune hemolytic anemia, antiphospholipid syndrome, corticosteroids, autoimmune neutropenia, systemic lupus erythematosus, idiopathic thrombocytopenia

## Abstract

Evans syndrome is an autoimmune disorder characterized by the simultaneous or sequential occurrence of autoimmune hemolytic anemia and idiopathic thrombocytopenic purpura (ITP) with or without neutropenia. It can occur idiopathically or secondary to autoimmune or malignant processes. We are presenting a case of ITP with concurrent chronic hemolytic anemia and positive markers for systemic lupus erythematosus with no prior diagnosis of any rheumatological disorder.

## Introduction

Evans syndrome is a rare autoimmune disorder first described in 1951 [1], where there is a simultaneous or sequential occurrence of autoimmune hemolytic anemia (AIHA) and idiopathic thrombocytopenic purpura (ITP) [2]. Autoimmune neutropenia can also occur [2]. It is estimated that it occurs in 7% of AIHA and 3% of ITP cases [3]. It occurs at a mean age of 55 ± 33 years, with female predominance [3]. It is important to exclude other etiologies such as anemia, thrombocytopenia like thrombotic microangiopathies, vitamin deficiencies, infections, and myelodysplastic syndrome. After confirming the diagnosis, it is prudent to determine whether it is primary or secondary Evans syndrome. The most common causes of secondary Evans syndrome are chronic lymphocytic leukemia (CLL), non-Hodgkin's lymphoma (NHL), and systemic lupus erythematosus (SLE) [2,3]. Rarely, it presents as the sole manifestation of a disease. Here, we present a case of Evans syndrome in the context of incomplete SLE.

## Case presentation

**Figure 1:**
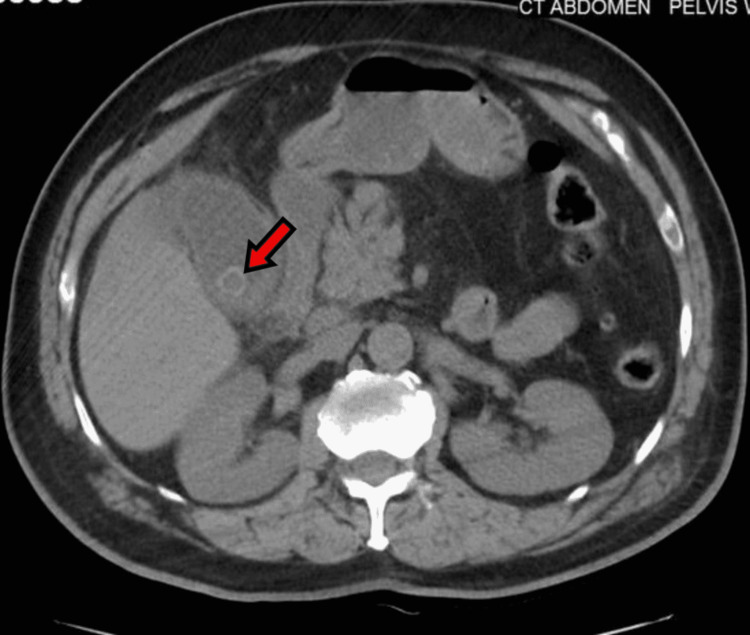
Cholelithiasis

**Figure 2:**
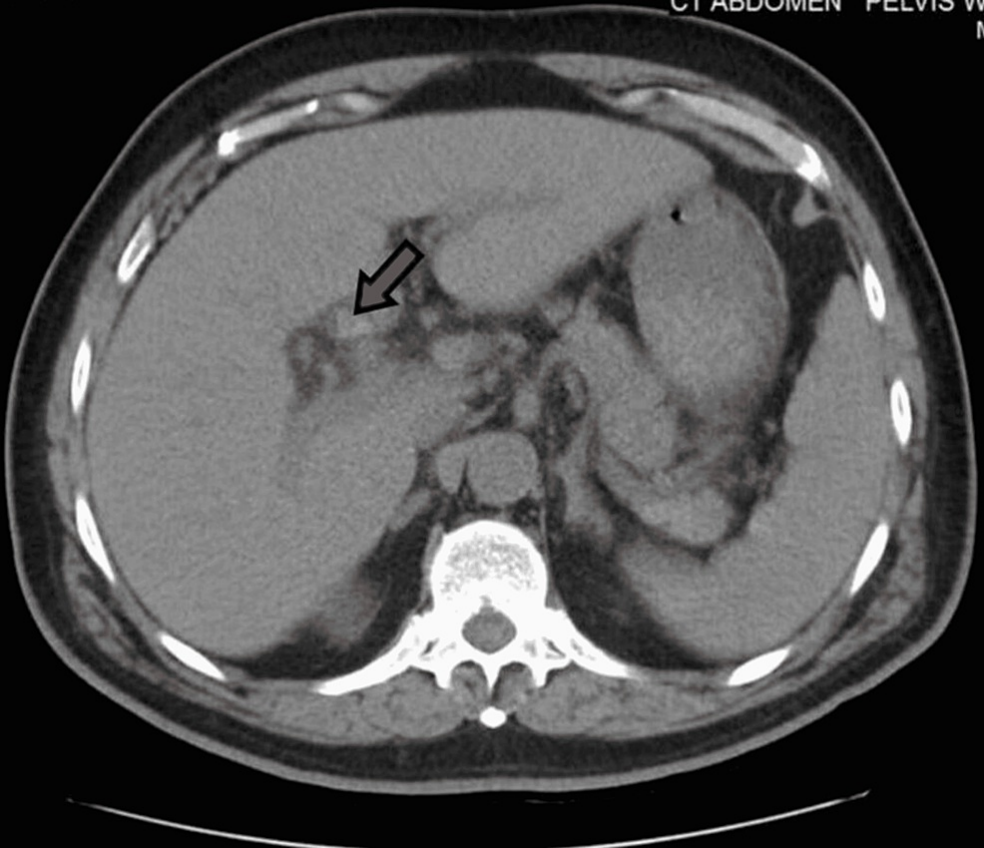
Extensive choledocholithiasis

A 71-year-old male with a past medical history of hypertension, hyperlipidemia, benign prostatic hyperplasia, and anemia presented to the ER referred by his primary medical doctor (PMD) for low platelets. He sought medical attention earlier at his PMD’s office for right upper quadrant pain and yellowish discoloration of the skin. He also mentioned having tarry stools for the last couple of days. The patient was afebrile and hemodynamically stable when he arrived at the ER and was completely coherent with no focal neuro deficit. Physical examination was noncontributory except for yellowish skin and icteric sclera and right upper quadrant tenderness. The rectal exam was positive for melena. Workup (Table 1) was significant for normal white blood cell count, anemia, severe thrombocytopenia, acute kidney injury, and prolonged activated partial thromboplastin time. Ultrasound of the abdomen and pelvis showed cholelithiasis and dilated extrahepatic duct system with multiple calculi in the common bile duct (largest was 5 mm). CT of the abdomen and pelvis confirmed the findings (Figures 1, 2), in addition to mild hepatosplenomegaly.

**Table 1:** Laboratory results on admission

Laboratory workup	Results	Reference range
White cell count	5.3 K/UL	4-11.2
Hemoglobin	10.0 g/dl	13.7-17.5
Platelets	9 K/UL	15-400
Blood urea nitrogen	32 mg/dl	7-18
Creatinine	1.6 mg/dl	0.7-1.3
Prothrombin time	17.2 seconds	12-14.8
International normalized ratio	1.4	0.9-1.12
Activated partial thromboplastin time	101.4 seconds	22.8-36.5
Fibrinogen	767 mg/dl	212-467.8
D-dimer	2.61 mcg/ml FEU	0-0.52
Iron	45 mcg/dl	65-175
Total iron-binding capacity	179 mcg/l	250-450
% saturation	25%	20-50
Ferritin	357 ng/ml	22-322
Folate	17.7 ng/ml	1.1-20
Vitamin B12	620 pg/ml	211-911
Retic count	1.72%	0.9-2.5
Haptoglobin	297 mg/dl	43-212
Lactate dehydrogenase	198 U/L	87-241

**Figure 3:**
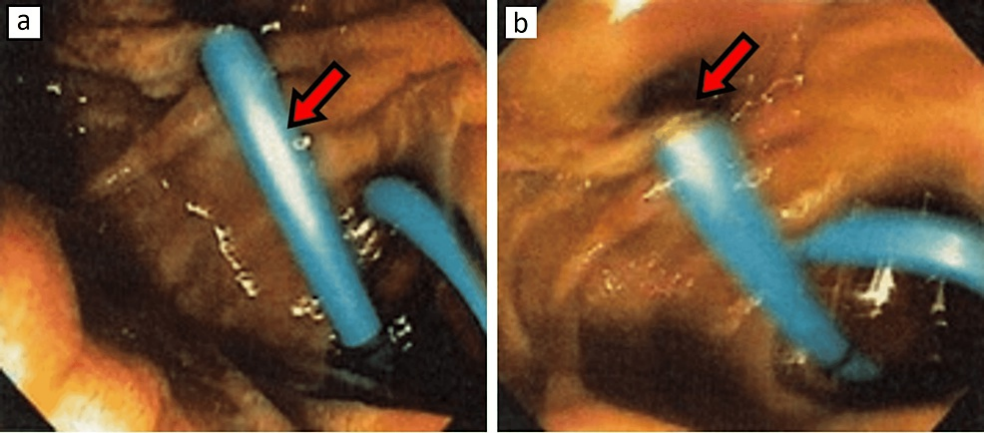
Red arrows in images A and B showing a 10 Fr x 7 cm double pigtail stent placed into the bile duct

A peripheral smear was taken, which showed neither spherocytes nor schistocytes. Giant platelets were seen with mild clumping, as read by the pathologist and hematologist. The patient was given a total of four units of platelets during the first 24 hours but the platelet count did not improve (platelets the next day were 17 K/UL). Creatinine improved on fluids to 0.9 mg/dl. Since thrombotic thrombocytopenic purpura was ruled out on peripheral smear and the patient was not septic clinically, the hematologist recommended starting prednisone at 1 mg/kg/day to treat immune thrombocytopenic purpura. His platelets steadily improved and reached 154 K/UL on day seven of admission (Table 2) with steroids. Endoscopic retrograde cholangiopancreatography (ERCP) was performed and a pigtail stent was placed by the gastroenterologist for treatment of choledocholithiasis (Figure 3).

**Table 2:** Platelet count from day one to day seven of admission The patient was given steroids on day two of admission.

Days	1	2	3	4	5	6	7
Platelet count	9 K/UL	17 K/UL	-	-	103 K/UL	-	154 K/UL

**Figure 4:**
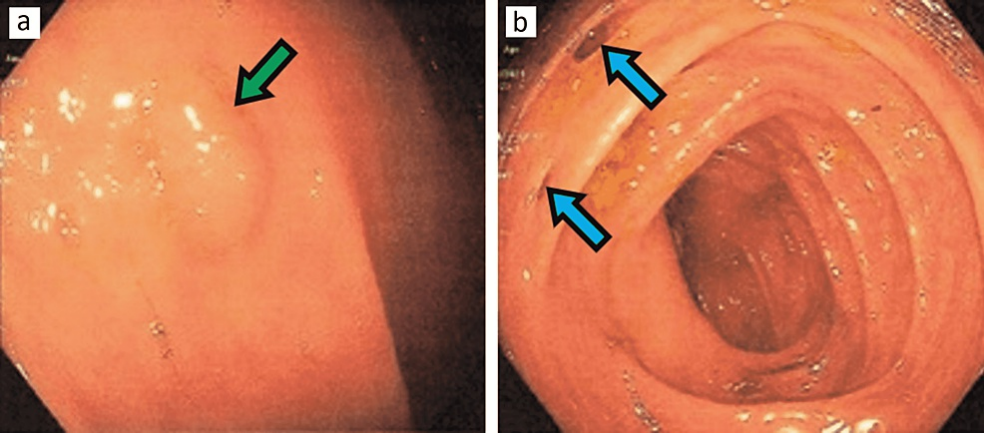
Green arrow in image A showing a 3 mm sessile polyp and blue arrows in image B showing small and large diverticulosis

Further workup included a viral panel (hepatitis A, B, and C profile and HIV screen), which was negative; antinuclear antibody (ANA), which turned out to be positive with 1:80 titer; anti-double-stranded DNA, which was negative; direct antiglobulin test (DAT), which was positive for warm antibodies; and mixing studies, which did not correct after mixing with normal plasma (activated partial thromboplastin time = 156; after mixing studies = 194). The patient’s hemoglobin was stable throughout the admission. Lupus anticoagulant was positive and anticardiolipin (>150 antiphospholipid antibodies) was highly positive on two separate occasions 12 weeks apart. The patient never had any thrombotic event in his life. The patient had a bone marrow biopsy and flow cytometry during admission, in addition to immunoglobulin levels, which were all unrevealing. He had chronic anemia, the etiology of which was unknown. He had recurrent choledocholithiasis in the past. Colonoscopy was performed before discharge, which showed diffuse diverticulosis and grade one internal hemorrhoids (Figure 4).

He was discharged on a tapering dose of steroids and was advised to follow up with a hematologist and gastroenterologist on an outside basis. Follow up visit showed a stable platelet count at around 78 K/UL with no signs of bleeding. Steroids were tapered off over three months with no evidence of relapse.

## Discussion

This is a case of a 71-year-old male who presented with choledocholithiasis and low platelet count. He had a history of chronic anemia with unknown etiology. An extensive workup was done on the patient during admission, including a peripheral smear that ruled out thrombotic thrombocytopenic purpura. Lab work ruled out sepsis and disseminated intravascular coagulation. Further workup including an extensive history of recent drug use, a viral panel including hepatitis A/B/C, HIV, Epstein-Barr virus (EBV), cytomegalovirus (CMV) polymerase chain reaction (PCR) and titers, bone marrow biopsy, and flow cytometry ruled out any blood dyscrasia. The patient improved on steroids. ITP diagnosis was reached after extensive negative workup. On the other hand, the patient had positive DAT, although parameters of acute hemolysis were absent like high lactate dehydrogenase (LDH), indirect bilirubin, low haptoglobin, and high retic count. Evidence of chronic hemolysis like recurrent choledocholithiasis and hepatosplenomegaly was present. In the absence of other sound explanations for his anemia, chronic AIHA is the plausible diagnosis for his chronic hemolysis in the presence of positive DAT. The sequential presence of both cytopenias makes Evans syndrome a proper diagnosis for the patient.

Most case reports and case series report a lapse time between both diagnoses of around three years [3]. The chronic nature of our patient’s AIHA makes it difficult to determine the exact lapse time between both cytopenia diagnoses. The positivity of ANA, lupus anticoagulant, and anticardiolipin ruled out the idiopathic nature of the syndrome. On the other hand, the patient did not meet the full criteria of either SLE or antiphospholipid syndrome. He rather met the criteria for incomplete SLE as per the contemporary American College of Rheumatology criteria with the only manifestation of Evans syndrome.

Around half of the cases of Evans syndrome occur idiopathically but the other half occur secondary to other systemic illnesses or malignancies. Around 15% of Evans syndrome cases occur in the context of NHL and 25% occur in the context of CLL. It is associated with autoimmune diseases in 18% of the cases [2,3].

There is no evidence-based treatment for Evans syndrome in light of its rarity. Many treatment modalities have been used with varying success rates. The first-line treatment is corticosteroids. Usually, prednisone is given at a dose of 1 mg/kg over three to four weeks [4] with an abrupt or slow tapper [5]. The initial response rate for both AIHA and ITP is 80%, but the one-year remission rate is 22-30% for ITP and 33% for AIHA [6]. Second-line treatment should be considered in severe case presentations, non-responders to first-line treatment, and to patients who require treatment for a longer duration on steroids to avoid its side effects [7]. Intravenous immunoglobulin (IVIG) can be given at a dose of 1 g/kg on day one and then repeated on day three if needed [4], mostly for ITP if the patient is presenting with bleeding or a severely low platelet count of less than 30K [8]. There is no evidence of using IVIG for AIHA. It can be given alone or in combination with corticosteroids for synergistic effect [9]. The choice of the second-line treatment depends on the underlying cause. Some agents used are cyclophosphamide, cyclosporine, azathioprine, mycophenolate, hydroxychloroquine, danazol, and dapsone [7,10]. Rituximab can be used for idiopathic Evans syndrome or Evans syndrome associated with SLE with a very initial response rate reaching 82% and a long-term response rate of 64% [2,11].

Splenectomy is sought only if the thrombocytopenia is severe and not responding to treatment or if the anemia is refractory. The initial response rate reaches 78-85% and the long-term response rate is 42-62% [2]. Splenectomy should be avoided for SLE patients if they have positive antiphospholipid antibodies [7]. Chemotherapy treating the underlying malignancy is usually the treatment of choice for autoimmune cytopenia in the context of malignancies.

## Conclusions

This is a case of Evans syndrome that occurred sequentially with ITP occurring years after chronic hemolytic anemia manifestations. Although positive for ANA and antiphospholipid serology, the patient did not meet the full diagnostic criteria of SLE or antiphospholipid syndrome. A tapered dose of steroids improved the patient's platelet count, and he had no relapse upon follow-up and no clinical manifestation of any rheumatologically or malignant process was noted. The patient needs to be followed up on a long-term basis to screen for the occurrence of any sign or symptom that would drive the diagnosis to complete SLE.
